# Foot Anthropometry Measures in Relation to Treatment in Patients with Rheumatoid Arthritis: A Longitudinal Study

**DOI:** 10.3390/healthcare12161656

**Published:** 2024-08-20

**Authors:** Maria Gamez-Guijarro, Andres Reinoso-Cobo, Maria Jose Perez-Galan, Ana Belen Ortega-Avila, Laura Ramos-Petersen, Marcelino Torrontegui-Duarte, Gabriel Gijon-Nogueron, Eva Lopezosa-Reca

**Affiliations:** 1 Department of Nursing and Podiatry, Faculty of Health Sciences, University of Malaga, Arquitecto Francisco Peñalosa 3, Ampliación de Campus de Teatinos, 29071 Malaga, Spain; mgamez303@uma.es (M.G.-G.); andreicob@uma.es (A.R.-C.); anaortavi@uma.es (A.B.O.-A.); lauraramos.94@uma.es (L.R.-P.); m.torrontegui@uma.es (M.T.-D.); evalopezosa@uma.es (E.L.-R.); 2 Department of Rheumatology, Hospital Universitario Virgen de las Nieves, 18014 Granada, Spain; mariaj.perez.galan.sspa@juntadeandalucia.es; 3 IBIMA Plataforma BIONAND, 29010 Malaga, Spain

**Keywords:** rheumatoid arthritis, foot, treatments, deformity

## Abstract

Approximately 90% of patients with arthritis exhibit forefoot deformities, including deformities within the metatarsophalangeal and proximal interphalangeal joints. Current pharmacological treatment with Disease Modifying Antirheumatic Drugs (DMARDs) consists of two groups: synthetic drugs (sDMARDs) and biological drugs (bDMARDs). The objective of our study was to investigate foot anthropometry changes in RA patients based on the administered treatment over a five-year period Method: A longitudinal analysis was conducted with RA patients who were grouped based on their pharmacological treatment. The pharmacological treatment groups were categorized into (I) methotrexate (MTX), (II) MTX plus biological treatments (including all variables), (III) biological treatment alone, and (IV) a miscellaneous group comprising patients with diverse treatments, including patients for whom various drugs had failed or who had not achieved remission with pharmacological treatment. For the anthropometric measurements, a foot measurement platform validated by McPoil et al. was used. Post hoc analyses with Bonferroni correction were performed to identify pairwise differences between the treatment groups while controlling for Type I errors due to multiple comparisons. Results: In the period from 2018 to 2023, significant changes were observed in several foot measurements. For instance, the MTX group showed a statistically significant increase in left heel width (*p* = 0.026). The MTX group experienced a slight increase in left foot length, while the Biologics and MTX + Bio groups exhibited more substantial increases in both maximum medial arch height and midfoot width. Conclusions: Different RA treatments can have a significant impact on foot structure over a five-year period, showing notable changes in heel width and overall foot morphology. Combined treatments with MTX and biologics potentially offer better management of RA.

## 1. Introduction

Rheumatoid arthritis (RA) manifests as a chronic and inflammatory autoimmune disease with a degenerative progression and multifactorial etiology driven by immunological processes. Characteristic symptoms, such as joint pain and stiffness, result in limitations in performing daily activities [1]. This condition typically presents as a gradual-onset symmetrical polyarthritis, progressing to irreversible structural and functional damage [2], and leading to anatomical deformities that evolve in parallel with the disease course [1]. Although RA affects various areas of the body, the hands and feet are the most commonly affected regions [3]. This condition is significantly associated with increased morbidity and mortality, a decreased quality of life [4], and reduced functionality [5] and self-care abilities [6], as well as increased fatigue and gait alterations [7].

Involvement and pain in foot joints are distinctive features of early RA and are almost ubiquitous during disease progression [8], leading to significant physical and psychosocial deterioration [9]. Foot and ankle deformities have been described by subdividing the foot into regions (forefoot, midfoot, and hindfoot) as if they were independent deformities, but they are strongly associated. A change or alteration in one of these regions will impact the other two [10,11]. Approximately 90% of patients exhibit forefoot deformities, with the metatarsophalangeal and proximal interphalangeal joints being the most commonly referenced [12,13]. These deformities include conditions such as Hallux Abductus Valgus (HAV), a lateral deviation of the great toe at the metatarsophalangeal joint, often with bunion formation, which has a prevalence of 64%, and lesser toe deformities, which can be reducible or rigid, and have been described in up to 86% of patients, affecting one or multiple toes. The presence of these described alterations results in an increase in forefoot width [14]. Between 40 and 60% of cases involve the midfoot, the least studied and referenced region, which is often associated with advanced disease stages and a decrease in the medial longitudinal arch (MLA) [12]. Hindfoot and ankle involvement ranges from 30 to 60%, with valgus deviation being the most described hindfoot deformity [15] and deviation in the tibiotalar joint also having a high incidence in both newly diagnosed and long-standing patients [13].

Disease-related foot deformities cause a redistribution of foot pressures, typically shifting to the medial and hindfoot. This change is directly related to increased pain when standing, walking, or performing daily activities [16,17,18,19]. Gait alterations in patients with foot involvement are considered to be a functional adaptation or as compensation for pain and deformity, and include decreased walking speeds, increased double support phase time, increased support surface, limited range of motion, limited propulsion capacity of the forefoot, reduced step length, flattened MLA, loss of balance, etc. [18,20,21,22].

Current pharmacological treatment with Disease Modifying Antirheumatic Drugs (DMARDs) consists of two options: synthetic DMARDs (sDMARDs), such as metotrexato, sulfasalazine, leflunomide, and chloroquine, which aim to control the inflammatory process; and biological drugs (bDMARDs), such as tolicizumab, adalimumab, certolizumab, abatacept, and rituximab, which target the autoimmune response [23]. Initiating treatment in the early stages of the disease is crucial. The newest therapeutic strategy is based on two main objectives: controlling the inflammatory process (tight control) and disease activity (treat to target). These treatments can be used independently or in combination, with no first-line treatment being universally recommended as choices depend on the clinical presentation and progression of the patient [24].

To date, the evaluation of foot deformities in rheumatoid arthritis has focused on anthropometric differences [25]. The publications related to foot deformities caused by rheumatoid arthritis are descriptive retrospective studies that report deformities, affected joints, or pain locations [26]. However, there is a significant lack of longitudinal studies. Our study aims to understand how treatments over time affect these deformities, addressing this gap to provide a more accurate understanding of their evolution in RA patients. The objective of our study was to investigate foot anthropometry changes in RA patients based on the administered treatment over a five-year period.

## 2. Materials and Methods

### 2.1. Ethical Approval

This study was conducted in accordance with the Declaration of Helsinki and received approval from the Ethics Committees of the University of Malaga (CEUMA-91-06-05-2015-H) and the Andalusian Health Service (PEIBA ARC000118-07-2022).

### 2.2. Study Design

The study was a longitudinal analysis conducted between 2018 and 2023. Data collection occurred in two phases: from January 15 to June 15 in 2018, and from March 15 to July 15 in 2023.

### 2.3. Participants

In 2018, an observational study was conducted at the Rheumatology Service of the University Hospital Virgen de las Nieves in Granada, Spain. These patients were diagnosed by the rheumatology service at Hospital Virgen de las Nieves. The study involved a cohort of 246 patients experiencing foot pain, who were all diagnosed with rheumatoid arthritis (RA) according to the 2010 Rheumatoid Arthritis Classification Criteria endorsed by the American College of Rheumatology and the European League Against Rheumatism [27]. In 2023, the study was repeated with 206 of the original participants.

Exclusion criteria in 2018 included the presence of concomitant musculoskeletal diseases, diseases of the central or peripheral nervous system, or endocrine disorders (e.g., diabetes mellitus). In 2023, 40 participants were excluded for the following reasons: they refused to participate, were unable to stand or walk, had undergone foot and/or ankle surgery after June 2018, had conditions such as senile dementia or Alzheimer’s disease, underwent a change of residence, or died.

Patients meeting the inclusion criteria were contacted by the Rheumatology Service at the University Hospital Virgen de las Nieves. They were provided with an information sheet and invited to participate. Those who consented were interviewed and given detailed information about the study. All participants signed informed consent forms prior to their interviews.

Pharmacological treatment data were categorized into (I) MTX, (II) MTX plus biological treatments (including all variables), (III) biological treatment alone, and (IV) a miscellaneous group comprising patients with diverse treatments, including patients for whom various drugs had failed or who had not achieved remission with pharmacological treatment.

**Figure 1:**
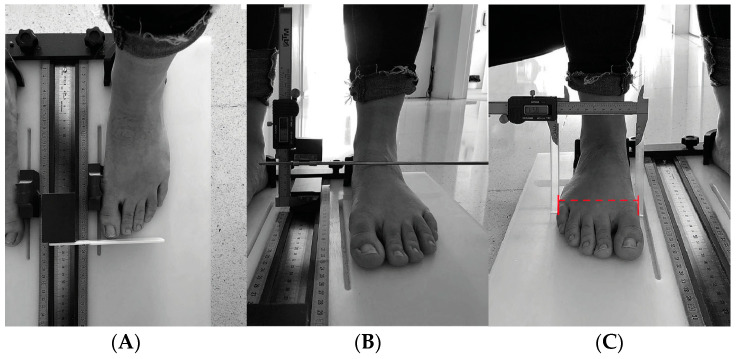

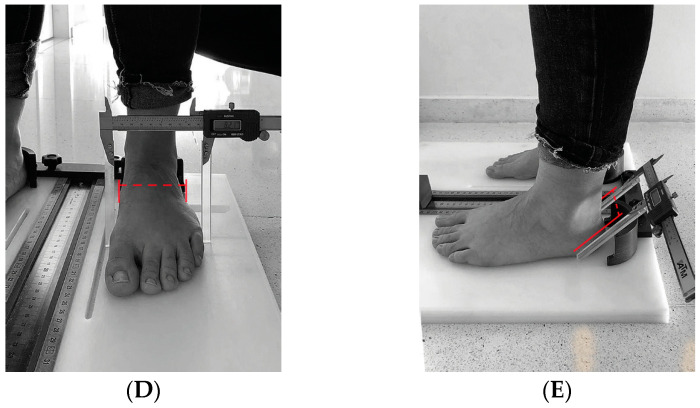
Protocol Mc Poil. (**A**) Length of foot, (**B**) maximum height medial arch, (**C**) forefoot width, (**D**) midfoot width, (**E**) heel width.

For the anthropometric measurements, a foot measurement platform validated by McPoil et al. [28] was used. This platform measured foot length, midfoot width, forefoot width, hindfoot width, and midfoot height under both load-bearing and non-load-bearing conditions. In our case, we decided to analyze the foot during load bearing, as it provides a more realistic measurement of the foot in its daily activities such as standing or walking. Each participant stood on the platform for the load-bearing measurement and then sat down for the non-load-bearing measurement. In both cases, participants stood in a relaxed position, looking straight ahead, with their weight evenly distributed between both feet (Figure 1).

Measurements were taken with the feet of the participants placed in the heel cups of the measurement platform, with the heels positioned as far back as possible and the first metatarsal heads against the surface boundary. One of the researchers (A.R.C.) conducted a repeatability and reliability analysis of this procedure with 30 participants, achieving an intraclass correlation coefficient (ICC) for the instrument ranging from 0.96 to 0.98.

The selection of the 30 subjects for the assessment of the intraclass correlation coefficients (ICCs) was based on the need to have a representative sample to assess the repeatability and reliability of the anthropometric foot measurements. This number of subjects is commonly used in validation studies and is considered adequate to provide an accurate estimate of the ICC [29]. The sample of 30 subjects was randomly selected from the study participants, ensuring that they represented the variability observed in the study population.

### 2.4. Sample Size

A power of 90% and a confidence level of 95% were used for the sample size to ensure that the study had the ability to detect significant differences in anthropometric foot measurements at a significance level of 5%. Calculations indicated that a sample of 206 patients would provide adequate statistical power to detect clinically significant changes in the measures evaluated.

The initial sample size of 246 patients was selected based on the prevalence of foot deformities in patients with rheumatoid arthritis. We considered this initial sample to be representative of the target population and adequate for the proposed longitudinal evaluation. In the follow-up conducted in 2023, 206 patients were included from the initial 246.

### 2.5. Statistical Analysis

Descriptive statistics of the variables were obtained, and the Kolmogorov–Smirnov test was utilized to assess the normality of the data distribution, ensuring the appropriateness of subsequent parametric tests. Between-group differences in variables at each time point were explored by ANCOVA, using baseline data as a covariate. The ANCOVA test was applied to control for potential confounding variables, thus providing a more accurate understanding of the effects under investigation. Within-group differences were examined with paired Student’s *t*-tests. Differences in demographic and baseline characteristics among the groups receiving different pharmacological treatments were assessed using one-way ANOVA. Post hoc analyses with Bonferroni correction were performed to identify pairwise differences between treatment groups while controlling for Type I error due to multiple comparisons.

For the assessment of the ICC, several underlying assumptions were evaluated. The normality of the data was checked using the Kolmogorov–Smirnov test, and the results confirmed that the distribution of the anthropometric foot measurements did not significantly deviate from a normal distribution. The homogeneity of the variances was assessed with Levene’s test, showing that the variances were homogeneous among the different measurements, thus validating the use of the ICC in this context. The mixed-effects model of the ICC was applied, which is appropriate for assessing interobserver reliability in repeated measures studies, as it accounts for both between-subject variability and measurement error. The ICC values obtained (ranging from 0.96 to 0.98) indicate the excellent reliability of the measurements.

For all tests, a two-tailed *p* value of <0.05 was considered statistically significant. All statistical analyses were conducted using SPSS v.26, a comprehensive software package that facilitated the management and analysis of the complex data sets involved in this research.

## 3. Results

The study sample comprised 206 patients with an average age of 58.32 years (SD 12.43) and an average disease duration of 15.28 years (SD 10.38); 76% were female and 24% were male, and the disease activity was DAS 28 2.7 (0.38) in 2018 and 2.1 (0.45) in 2023 (*p* = 0.237) without changes between activity groups (Table 1). In 2018, according to the activity level, 20.39% of the patients were in remission, 44.66% had low activity, 29.13% had moderate activity, and 5.83% had high activity. In 2023, 46.60% were in remission, 17.48% had low activity, 30.10% had moderate activity, and 5.83% had high activity.

Within the miscellaneous treatment group, the activity levels remained similar between 2018 and 2023, although a significant difference was observed in moderate activity (*p* = 0.04), while the differences in the other activities were not significant (*p* = 0.390).

The study analyzed changes in foot anthropometry among rheumatoid arthritis (RA) patients categorized into four treatment groups. Over the period from 2018 to 2023, significant changes were observed in several foot measurements. For instance, the MTX group showed a statistically significant increase in left heel width (*p* = 0.026). The foot length measurements did not show significant increases. In some cases, decreases in right foot length and variations in left foot length were observed over a five-year period. Across all groups, there were notable increases in midfoot width, forefoot width, and heel width, with varying degrees of significance. The MTX group had a slight increase in left foot length, while the Biologics and MTX + Bio groups exhibited more substantial increases in both the maximum medial arch height and midfoot width. These findings highlight the impact of different RA treatments on foot structure over time (see Table 2 for detailed results).

## 4. Discussion

The present study aimed to analyze the impact of different pharmacological treatments on foot anthropometry in patients with rheumatoid arthritis (RA) over a five-year period. To address this gap and provide a more precise understanding of the evolution of foot deformities in RA patients, we recruited a cohort of 246 patients from various studies at the University Hospital Virgen de las Nieves.

It is important to note that not all of the results reached statistical significance. In particular, variations in left foot length in the MTX treatment group were not statistically significant (*p* > 0.05). The lack of significance may be due to the intrinsic variability of anthropometric measurements and sample size.

In addition, variations between the left and right feet were observed in several anthropometric measurements. In some patients, the right foot showed a greater decrease in length compared to the left foot. These differences may be attributed to several factors, including foot dominance and differential loading during gait [30,31].

The literature suggests that left-right foot differences are common in patients with rheumatoid arthritis due to asymmetric load distribution and unequal disease progression [32]. Therefore, it is crucial to consider these variations when interpreting results and designing treatments.

Combined pharmacological treatments are proposed to achieve faster and more effective disease remission, thereby enhancing overall treatment efficacy. However, achieving remission, as reflected by a DAS28 score on the designated scale, does not solely determine the progression of foot deformities [33]. Foot morphology significantly influences patient mobility and quality of life. It is noteworthy that current methods for assessing disease activity often do not take foot joints into account. Given the obtained results, this issue warrants further investigation in future studies.

RA patients often experience foot deformities due to chronic inflammation and joint destruction, which can significantly impair their mobility and quality of life. Our study found that different treatment regimens had varying effects on foot anthropometry. During the five-year study period, significant changes were observed in several foot measurements. Specifically the MTX group, which showed a significant increase in left heel width (*p* = 0.026). The Biologics and MTX + Bio groups exhibited more substantial increases in maximum medial arch height and midfoot width. These changes indicate that combined treatments may offer better management of RA by positively affecting foot structure. This aligns with findings from previous studies, which indicate that effective RA management can mitigate the progression of joint damage, although deformities might still develop due to the chronic nature of the disease [34,35].

The observed changes in foot length, midfoot width, forefoot width, and heel width across all treatment groups are consistent with the literature, which documents widespread foot alterations in RA patients [36,37]. Notably, studies have shown that biologics can significantly reduce disease activity and prevent joint destruction, thus improving foot function and morphology [38,39]. However, despite the overall benefits of biologics, our findings suggest that structural changes in the foot continue to occur, underscoring the need for ongoing monitoring and adjustment of treatment plans.

Regarding the relationship of anthropometric measures with the demographic characteristics of the patients, we found that our results indicate that patients with high activity levels (DAS28) tend to show a greater decrease in foot length and an increase in forefoot width, which is consistent with the findings of Turner et al. [19]. In a similar longitudinal study, variations in anthropometric foot measurements were observed in patients with rheumatoid arthritis, supporting our findings of changes in foot length.

Based on the obtained results, we can infer that combined treatments with MTX and biologics are not only effective in controlling disease activity but also contribute to the modification of foot structure, which could have positive implications for patient mobility and quality of life. These findings highlight the importance of an integrated treatment approach that includes regular assessment of foot morphology and the adaptation of personalized interventions, such as orthotic use, to manage the long-term effects of RA on foot health.

The clinical implications of these findings are substantial. Foot deformities in RA patients often lead to altered gait patterns, increased pain, and a reduced ability to perform daily activities. These changes can be exacerbated by improper footwear and lack of supportive interventions such as orthotics [34,40]. Therefore, incorporating comprehensive foot care, including regular podiatric assessments and personalized orthotic support, could be crucial in managing the long-term effects of RA on foot health.

As for limitations, the study relied on anthropometric measurements to evaluate foot deformities. While these measurements are objective, they may not fully capture the functional impact on the daily lives of patients and may not correspond with information provided by radiological tests. Including functional assessments, patient-reported outcomes, and quality of life measures could provide a more accurate reflection of individual realities. Current methods for assessing disease activity, such as the DAS28, generally do not account for the foot joints, which are significantly affected in RA. This deficiency can lead to an incomplete evaluation of disease activity and treatment efficacy concerning the foot. Future studies should incorporate more specific assessments of foot joints.

Another of the limitations of our study is that external factors such as the type of footwear worn by the participants were not considered. For future studies, we recommend including a detailed assessment of the type of footwear worn by the participants or the inclusion of a control group.

The study evaluated the impact of different pharmacological treatments, but there could be variability in treatment adherence, dosages, and combinations that were not fully controlled. This variability can influence the outcomes and may not be representative of a standardized treatment regimen. Selection bias might be present since the study participants were recruited from a hospital setting, potentially excluding RA patients who do not seek hospital care regularly. Additionally, the retrospective nature of some of the data collection could introduce recall bias.

## 5. Conclusions

This study highlights the significant impact of different RA treatments on foot structure over five years, showing notable changes in heel width and overall foot morphology. Combined treatments with MTX and biologics potentially offer better management of RA, emphasizing the need for integrated treatment approaches. Longitudinal assessments are crucial for understanding the long-term effects of these treatments on foot deformities.

## Figures and Tables

**Table 1:** Characteristic of the sample.

	2018				2023			
	Mean	CI 95%		SD	Mean	CI 95%		SD
Age (years)	56.59	54.47	58.72	10.87	61.88	59.74	64.03	10.97
Weight (kg)	71.92	68.85	74.99	15.70	71.36	69.8	73.1	13.67
Height (cm)	163.15	161.69	164.60	7.44	162.65	161.24	164.06	7.20
Evolution (years)	13.84	11.86	15.83	10.17	20.04	17.78	22.30	11.56
ESR	19.10	15.64	22.56	17.70	18.25	14.96	21.54	16.83
CRP	1.20	0.82	1.58	1.94	3.61	2.30	4.91	6.68
DAS28	2.15	1.96	2.34	0.97	2.66	2.45	2.86	1.04

Abbreviations: CRP (C reactive protein); ESR (erythrocyte sedimentation rate); DAS28 (disease activity score); SD (standard deviation); CI (Confidence Interval).

**Table 2:** Anthropometric changes in foot measurements of RA patients by treatment group from 2018 to 2023.

		2018	2023	
	Type of Treatment (N)	Mean (SD)	Mean (SD)	*p* Value
Length right foot	MTX (42)	240.48 (9.7)	239.24 (9.8)	0.888
	bDMARDs (115)	245.09 (13.2)	243.26 (13.6)	
	MTX+ bDMARDs (33)	243.50 (14.9)	242.10 (15)	
	Other (16)	247.56 (13.4)	246 (14.4)	
Length left foot	MTX (42)	242.29 (10.6)	242.48 (10.5)	0.05
	bDMARDs (115)	246.04 (13.5)	245.06 (13)	
	MTX+ bDMARDs (33)	245.7 (15.6)	245.75 (14.7)	
	Other (16)	248.33 (14.3)	245.67 (15.6)	
Maximum height Medial Arch Right	MTX (42)	51.94 (5.3)	54.58 (6.9)	0.641
	bDMARDs (115)	54.34 (6.5)	56.10 (8.3)	
	MTX+ bDMARDs (33)	54.17 (4.8)	54.54 (7.5)	
	Other (16)	50.65 (5.9)	53.02 (7.7)	
Maximum height Medial Arch Left	MTX (42)	53.77 (5.3)	55.50 (7.9)	0.447
	bDMARDs (115)	56.51 (6.3)	58.35 (8.5)	
	MTX+ bDMARDs (33)	56.56 (4.7)	56.85 (8.3)	
	Other (16)	52.09 (7.2)	56.64 (7.5)	
Midfoot width Right	MTX (42)	74.48 (5.8)	77.01 (5.5)	0.56
	bDMARDs (115)	78.68 (6.4)	81.77 (6.3)	
	MTX+ bDMARDs (33)	79.17 (5.8)	82.98 (6)	
	Other (16)	77.74 (5.7)	80.38 (8.2)	
Midfoot width Left	MTX (42)	75.71 (5.3)	76.19 (6.1)	0.483
	bDMARDs (115)	79.58 (6.7)	79.90 (6.6)	
	MTX+ bDMARDs (33)	79.72 (5.1)	80.98 (5.4)	
	Other (16)	78.79 (5.5)	80.33 (7.0)	
Forefoot width right	MTX (42)	89.40 (5.6)	92.44 (5.6)	0.345
	bDMARDs (115)	92.47 (5.5)	96.47 (6.2)	
	MTX+ bDMARDs (33)	92.15 (4.6)	95.92 (6.3)	
	Other (16)	90.84 (6.2)	96.30 (6.9)	
Forefoot width Left	MTX (42)	89.06 (4.9)	92.99 (5.5)	0.958
	bDMARDs (115)	92.33 (6.6)	96.39 (6.2)	
	MTX+ bDMARDs (33)	91.16 (4.7)	95.09 (5.8)	
	Other (16)	90.01 (7)	94.63 (8.7)	
Heel width Right	MTX (42)	64.69 (4.5)	66.77 (6.8)	0.867
	bDMARDs (115)	67.97 (4.9)	70.37 (5.7)	
	MTX+ bDMARDs (33)	67.12 (5.1)	70.07 (5.9)	
	Other (16)	66.74 (4.7)	69.16 (6.8)	
Heel width Left	MTX (42)	64.64 (5.3)	67.64 (7.1)	0.026
	bDMARDs (115)	68.20 (5)	70.14 (5.3)	
	MTX+ bDMARDs (33)	68.50 (5.1)	71.43 (5.6)	
	Other (16)	65.17 (5.1)	70.11 (6)	

Abbreviations: Methotrexate (MTX), Biologics drugs (bDMARDs), Methotrexate combined with Biologics (MTX + Bio).

## Data Availability

The data presented in this study are available on request from the corresponding author.

## References

[1] Nieves A.T., Holguera R.M., Gómez A.P., de Mon-Soto M. (2017). Artritis reumatoide. Medicine.

[2] Sharif K., Sharif A., Jumah F., Oskouian R., Tubbs R.S. (2018). Rheumatoid arthritis in review: Clinical, anatomical, cellular and molecular points of view. Clin. Anat..

[3] Kourilovitch M., Galarza-Maldonado C., Ortiz-Prado E. (2014). Diagnosis and classification of rheumatoid arthritis. J. Autoimmun..

[4] Reinoso-Cobo A., Gijon-Nogueron G., Caliz-Caliz R., Ferrer-Gonzalez M.A., Vallejo-Velazquez M.T., Morales-Asencio J.M., Ortega-Avila A.B. (2020). Foot health and quality of life in patients with rheumatoid arthritis: A cross-sectional study. BMJ Open.

[5] Munchey R., Pongmesa T. (2018). Health-Related Quality of Life and Functional Ability of Patients with Rheumatoid Arthritis: A Study from a Tertiary Care Hospital in Thailand. Value Health Reg. Issues.

[6] Zuidema R., Repping-Wuts H., Evers A., Van Gaal B., Van Achterberg T. (2015). What do we know about rheumatoid arthritis patients’ support needs for self-management? A scoping review. Int. J. Nurs. Stud..

[7] Schmiegel A., Rosenbaum D., Schorat A., Hilker A., Gaubitz M. (2008). Assessment of foot impairment in rheumatoid arthritis patients by dynamic pedobarography. Gait Posture.

[8] Yano K., Ikari K., Inoue E., Sakuma Y., Mochizuki T., Koenuma N., Tobimatsu H., Tanaka E., Taniguchi A., Okazaki K. (2018). Features of patients with rheumatoid arthritis whose debut joint is a foot or ankle joint: A 5479-case study from the IORRA cohort. PLoS ONE.

[9] Zhang C., Wu X., Yuan Y., Xiao H., Li E., Ke H., Yang M., Zhu X., Zhang Z. (2022). Effect of solution-focused approach on anxiety and depression in patients with rheumatoid arthritis: A quasi-experimental study. Front. Psychol..

[10] Loveday D.T., Jackson G.E., Geary N.P. (2012). The rheumatoid foot and ankle: Current evidence. Foot Ankle Surg..

[11] Brooks F., Hariharan K. (2013). The rheumatoid forefoot. Curr. Rev. Musculoskelet. Med..

[12] Stolt M., Suhonen R., Leino-Kilpi H. (2017). Foot health in patients with rheumatoid arthritis—A scoping review. Rheumatol. Int..

[13] Lee S.W., Kim S.Y., Chang S.H. (2019). Prevalence of feet and ankle arthritis and their impact on clinical indices in patients with rheumatoid arthritis: A cross-sectional study. BMC Musculoskelet. Disord..

[14] Rome K., Gow P.J., Dalbeth N., Chapman J.M. (2009). Clinical audit of foot problems in patients with rheumatoid arthritis treated at Counties Manukau District Health Board, Auckland, New Zealand. J. Foot Ankle Res..

[15] Borman P. (2012). Foot Problems in a Group of Patients with Rheumatoid Arthritis: An Unmet Need for Foot Care. Open Rheumatol. J..

[16] van der Leeden M., Steultjens M.P.M., Terwee C.B., Rosenbaum D., Turner D., Woodburn J., Dekker J. (2008). A systematic review of instruments measuring foot function, foot pain, and foot-related disability in patients with rheumatoid arthritis. Arthritis Care Res..

[17] Onodera T., Nakano H., Homan K., Kondo E., Iwasaki N. (2019). Preoperative radiographic and clinical factors associated with postoperative floating of the lesser toes after resection arthroplasty for rheumatoid forefoot deformity. BMC Musculoskelet. Disord..

[18] Khazzam M., Long J.T., Marks R.M., Harris G.F. (2007). Kinematic changes of the foot and ankle in patients with systemic rheumatoid arthritis and forefoot deformity. J. Orthop. Res..

[19] Turner D., Helliwell P., Siegel K.L., Woodburn J. (2008). Biomechanics of the foot in rheumatoid arthritis: Identifying abnormal function and the factors associated with localised disease ‘impact’. Clin. Biomech..

[20] Turner D.E., Woodburn J., Helliwell P.S., Cornwall M.W., Emery P. (2003). Pes planovalgus in RA: A descriptive and analytical study of foot function determined by gait analysis. Musculoskelet. Care.

[21] Dubbeldam R., Baan H., Nene A.V., Drossaers-Bakker K.W., van de Laar M.A.F.J., Hermens H.J., Buurke J.H. (2013). Foot and ankle kinematics in rheumatoid arthritis: Influence of foot and ankle joint and leg tendon pathologies. Arthritis Care Res..

[22] Schmiegel A., Vieth V., Gaubitz M., Rosenbaum D. (2008). Pedography and radiographic imaging for the detection of foot deformities in rheumatoid arthritis. Clin. Biomech..

[23] Radner H., Smolen J.S., Aletaha D. (2014). Remission in rheumatoid arthritis:Benefit over low disease activity in patient-reported outcomes and costs. Arthritis Res. Ther..

[24] Kuijper T.M., Lamers-Karnebeek F.B., Jacobs J.W., Hazes J.M., Luime J.J. (2015). Flare Rate in Patients with Rheumatoid Arthritis in Low Disease Activity or Remission When Tapering or Stopping Synthetic or Biologic DMARD: A Systematic Review. J. Rheumatol..

[25] Sanchez-Castillo J.A., Reinoso-Cobo A., Gijon-Nogueron G., Caliz-Caliz R., Exposito-Ruiz M., Ramos-Petersen L., Ortega-Avila A.B. (2021). Symmetry Criterion for Patients with Rheumatoid Arthritis of the Foot: A Cross-Sectional Study. Int. J. Environ. Res. Public Health.

[26] van der Leeden M., Steultjens M.P., van Schaardenburg D., Dekker J. (2010). Forefoot disease activity in rheumatoid arthritis patients in remission: Results of a cohort study. Arthritis Res. Ther..

[27] Aletaha D., Neogi T., Silman A.J., Funovits J., Felson D.T., Bingham C.O., Birnbaum N.S., Burmester G.R., Bykerk V.P., Cohen M.D. (2010). 2010 Rheumatoid arthritis classification criteria: An American College of Rheumatology/European League against Rheumatism collaborative initiative. Arthritis Rheum..

[28] McPoil T.G., Vicenzino B., Cornwall M.W., Collins N. (2009). Can foot anthropometric measurements predict dynamic plantar surface contact area?. J. Foot Ankle Res..

[29] Koo T.K., Li M.Y. (2016). A Guideline of Selecting and Reporting Intraclass Correlation Coefficients for Reliability Research. J. Chiropr. Med..

[30] Biscontini D., Bocci E.B., Gerli R. (2009). Analysis of foot structural damage in rheumatoid arthritis: Clinical evaluation by validated measures and serological correlations. Reumatismo.

[31] Kuryliszyn-Moskal A., Kaniewska K., Dzięcioł-Anikiej Z., Klimiuk P.A. (2017). Evaluation of foot static disturbances in patients with rheumatic diseases. Reumatologia.

[32] Turner D.E., Woodburn J. (2008). Characterising the clinical and biomechanical features of severely deformed feet in rheumatoid arthritis. Gait Posture.

[33] Rantalaiho V., Kautiainen H., Korpela M., Hannonen P., Kaipiainen-Seppänen O., Möttönen T., Kauppi M., Karjalainen A., Laiho K., Laasonen L. (2014). Targeted treatment with a combination of traditional DMARDs produces excellent clinical and radiographic long-term outcomes in early rheumatoid arthritis regardless of initial infliximab. The 5-year follow-up results of a randomised clinical trial, the NEO-RACo trial. Ann. Rheum. Dis..

[34] Noguchi T., Hirao M., Tsuji S., Ebina K., Tsuboi H., Etani Y., Akita S., Hashimoto J. (2021). Association of Decreased Physical Activity with Rheumatoid Mid-Hindfoot Deformity/Destruction. Int. J. Environ. Res. Public Health.

[35] Takakubo Y., Wanezaki Y., Oki H., Naganuma Y., Shibuya J., Honma R., Suzuki A., Satake H., Takagi M. (2021). Forefoot Deformities in Patients with Rheumatoid Arthritis: Mid- to Long-Term Result of Joint-Preserving Surgery in Comparison with Resection Arthroplasty. Int. J. Environ. Res. Public Health.

[36] Woodburn J., Helliwell P.S. (1997). Foot problems in rheumatology. Br. J. Rheumatol..

[37] Scott D.L., Wolfe F., Huizinga T.W.J. (2010). Rheumatoid arthritis. Lancet.

[38] Fermoso R.B., Lozano M.R.M., Cordero M.N., Rincón C.M., García-Fernández P., Fernández M.L.G. (2024). Differences and Similarities in the Feet of Metatarsalgia Patients with and without Rheumatoid Arthritis in Remission. J. Clin. Med..

[39] Ramos-Petersen L., Nester C.J., Reinoso-Cobo A., Nieto-Gil P., Ortega-Avila A.B., Gijon-Nogueron G. (2020). A Systematic Review to Identify the Effects of Biologics in the Feet of Patients with Rheumatoid Arthritis. Medicina.

[40] Reina-Bueno M., Munuera-Martínez P.V., Pérez-García S., Vázquez-Bautista M.d.C., Domínguez-Maldonado G., Palomo-Toucedo I.C. (2021). Foot Pain and Morphofunctional Foot Disorders in Patients with Rheumatoid Arthritis: A Multicenter Cross-Sectional Study. Int. J. Environ. Res. Public Health.

